# Epigenetic Modification of the Leptin Promoter in Diet-Induced Obese Mice and the Effects of N-3 Polyunsaturated Fatty Acids

**DOI:** 10.1038/srep05282

**Published:** 2014-06-13

**Authors:** Wenwen Shen, Cui Wang, Lulu Xia, Chaonan Fan, Hua Dong, Richard J. Deckelbaum, Kemin Qi

**Affiliations:** 1 Nutrition Research Unit, Beijing Pediatric Research Institute, Beijing Children's Hospital, Capital Medical University, Beijing, China. No. 56 Nan-li-shi Road, Beijing 100045, China; 2 Institute of Human Nutrition, College of Physicians and Surgeons, Columbia University, New York, USA; 3 These authors contributed equally to this work.

## Abstract

We report evidence of a detailed epigenetic modification of the leptin promoter and the effects of n-3 polyunsaturated fatty acids (n-3 PUFAs), which is closely associated with the leptin gene transcription in obesity. In the adipose tissue of diet induced obese (DIO) mice, methylation of the CpG island and the binding of methyl-CpG-binding domain protein 2 (MBD2) and DNA methyltransferases (DNMTs) at the leptin promoter are increased and RNA Pol II is decreased. Additionally, histones H3 and H4 are hypoacetylated, lysine 4 of histone H3 (H3K4) is hypomethylated and the binding of histone deacetylases (HDACs) 1, 2 and 6 is increased at the leptin promoter in the DIO mice. These modifications may serve a feedback role to maintain leptin concentrations within a normal range. The regulation of leptin transcriptional expression by n-3 PUFAs is mediated, at least in part, by epigenetic targets, such as MBD2 and histone modifications.

In recent years, epigenetic modifications, such as DNA methylation and histone modifications, have received attention because accumulating evidence has linked the pattern of epigenetic modifications found at particular loci with the level of transcription of the corresponding genes^1^,^2^,^3^. DNA methylation, which occurs on the carbon-5 position of cytosine in CpG dinucleotides and is catalyzed by a family of DNA methyltransferases (DNMTs), has profound effects on gene expression by modifying the accessibility of DNA to transcription factors. It is well accepted that DNA methylation occurring on CpG islands is usually associated with gene silencing, whereas hypomethylation of non-CpG island-associated promoters deregulate gene expression^4^,^5^. The acetylation of histones H3 and H4 by histone acetyltransferases (HATs) induces an open chromatin conformation that allows the transcription machinery to access the promoter, leading to the activation of gene expression. Conversely, histone deacetylases (HDACs) reverse histone acetylation, resulting in a more compact chromatin environment in which transcription is repressed^4^,^5^,^6^,^7^. Histone methylation occurs at lysine and arginine residues and has both positive and negative effects on transcription depending on the site of methylation. For example, the methylation of lysine 4 of histone H3 (H3K4) usually correlates with gene activation, whereas the methylation of H3K9 or H3K27 is associated with transcriptional silencing^4^,^8^. Recent studies have revealed a key component of the epigenetic network controlling adipogenesis, food intake and energy homeostasis; the role of epigenetic modifications in human nutrition and obesity will be a fruitful area for further research^8^,^9^,^10^.

The cloning of the *ob* gene and identification of its product leptin has led to several new insights and has uncovered a new endocrine system regulating body weight. Leptin is mainly produced in adipose tissue and has been shown to play an important role in maintaining homeostatic control of adipose tissue mass and body weight by regulating food intake and energy expenditure through a variety of neural and endocrine mechanisms^11^,^12^. In diet-induced obesity (DIO), the gene expression of leptin and its circulating concentration are elevated with the absence of regulatory effects on body weight, indicating the development of leptin resistance^11^,^12^. So far, the molecular mechanisms underlying the abnormal expression of leptin in obesity remain unclear. Several studies have demonstrated that methylation of CpG island in the leptin promoter plays an important role in leptin expression during pre-adipocyte differentiation^13^,^14^ and may be involved in obesity-related leptin upregulation; however, these results have been inconsistent^15^,^16^,^17^. CpG methylation at gene promoters has been demonstrated to silence transcription not only by blocking transcription factor binding but also through the recruitment of the methyl-CpG-binding domain proteins (MBDs) which recruits HDAC and histone methyltransferase (HMT) complexes to the DNA causing histone deacetylation and the methylation of specific lysine residues^18^,^19^. Therefore, there is a need for further investigation of the epigenetic mechanisms that contribute to leptin expression and resistance in obesity.

Nutrients can modify physiological and pathological processes through altered gene expression, and epigenetic modifications are considered to be a key mechanism underlying the effects of nutrition on gene expression^1^,^3^. N-3 polyunsaturated fatty acids (n-3 PUFAs) function to reduce body fat and ameliorate pathological activities. These benefits have been attributed to the altered expression of a variety of genes that favor increased fat oxidation, reduced fat deposition, reduced food intake and the apoptosis of adipocytes^20^,^21^,^22^,^23^. The results from in vivo and in vitro studies have demonstrated that n-3 PUFAs are negatively associated with body leptin levels and reduce the expression of leptin^21^,^22^,^23^. Similar to the effects of other nutrients on the epigenetic modification of chronic disease-related genes^1^,^3^, n-3 PUFAs may affect epigenetic processes because methyl groups are required for the metabolism of docosahexaenoic acid (DHA)^24^. Our previous study demonstrated that the n-3 PUFAs in fish oil did not affect the CpG site methylation of the leptin promoter^17^. Thus, we hypothesized that n-3 PUFAs may function epigenetically by modulating the leptin promoter-associated proteins. In this study, using a high-fat DIO mouse model, we determined whether the binding of MBD2, the association of epigenetic modifying enzymes and the presence of histone modifications at the leptin promoter are associated with changes in leptin gene expression and determined the effects of n-3 PUFAs on these epigenetic features of the leptin gene.

## Methods

### Diets

Two high-fat diets (34.9% fat by wt., 60% kcal), the DIO diet and the n-3 PUFA DIO diet, were designed based on the high-fat diet formula (D12492) for DIO mice from Research Diets, Inc. (New Brunswick, NJ). These two DIO diets contained the same amount of lard as the main source of fat in each diet, which provided 89% of the total energy from fat. The DIO and n-3 PUFA DIO diets included sunflower oil or fish oil, respectively, as the PUFA source. In addition, a low-fat diet (4.3% fat by wt., 10% kcal) was also designed as a control with sunflower oil as the source of PUFAs and was based on the control diet formula (D12450B) from Research Diets, Inc. The fatty acid composition of all diets is shown in Table 1. The n-3 PUFAs accounted for 0.12% and 3.07% of the total fatty acids in the DIO and n-3 PUFA DIO diets, respectively. The diets were prepared by the Institute of Laboratory Animal Sciences at the Chinese Academy of Medical Sciences and stored at −20°C before use.

### Animals

Three- to four-week old C57BL/6J male mice were purchased from the Laboratory Animal Center of the Academy of Military Medical Sciences of China and housed at the animal facilities under a 12-hour (h) light/12-h dark cycle with cycles of air ventilation. After one week of recovery from transportation, the mice were fed the experimental diets for 14 weeks to induce obesity. For the DIO experiment, the mice were fed one of the two DIO diets or the control diet at 17:00 each day, and the body weights were measured weekly for 14 weeks. At the end of each feeding period, the 12-hour fasted mice (n = 12 in each group) were anesthetized by intraperitoneal injection of Avertin (125 mg/kg of 2,2,2-tribromoethanol, T-4840-2, Sigma-Aldrich Chemie GmbH, Steinheim, Germany) to obtain blood samples by heart puncture. The plasma was stored at −20°C for later analysis of the leptin concentration. After sacrificing each mouse, the epididymal fat was dissected free of the surrounding tissue, removed, wrapped in aluminum foil and frozen in liquid N_2_. Once an entire group of animals was harvested, the tissues were transferred to −80°C until the analysis was performed.

In order to investigate the epigenetic changes of the leptin promoter during development of obesity, another experiment was designed. Three to four-week old male mice were classified into two groups and fed with the DIO diet and the low-fat control diet respectively. The body weight was measured weekly to confirm that mice in the DIO group gained more body weight than those in the control group. At 4-, 8-, 12-, and 18-weeks after feeding respectively, the 12-hour fasted mice (n = 15 at each time point) from each of the two groups were sacrificed and the epididymal fat was dissected for use under the same procedure as the above mentioned.

All of the animal experiments were conducted from 08:00 to 12:00 in strict accordance with the recommendations in the Guide for the Care and Use of Laboratory Animals of National Administration Regulations on Laboratory Animals of China. The protocol was approved by the Committee on the Ethics of Animal Experiments of the Academy of Military Medical Sciences.

### Quantitative analysis of mRNA expression

Total RNA was extracted from mouse fat using TRIzol Reagent (cat. no. 15596-026, Invitrogen, Carlsbad, CA, USA), and cDNA was prepared from the total RNA using the SuperScript^TM^ III First-Strand Synthesis System for RT-PCR (cat. no. 18080-051, Invitrogen, Carlsbad, CA, USA), according to the procedures provided by the manufacturer. The leptin mRNA levels in the fat were measured using real-time quantitative RT-PCR with an ABI PRISM 7300 sequence detection system (Applied Biosystems, Foster City, CA, USA). The oligonucleotide primers and a TaqMan® probe for the leptin gene were designed and prepared by ABI Applied Biosystems (Foster City, CA, USA). The co-amplification of mouse GAPDH mRNA, an invariant internal control, was performed in all the samples because this house-keeping gene expression is little affected by dietary fat. The assays were performed in triplicate, and the results were normalized to the GAPDH mRNA levels using the 2^–ΔCT^ method.

### Bisulfite conversion and sequencing

The examined leptin promoter region includes nucleotides (nts) 29009221-29010220 and spans 16 CpGs within nts -321 to -1 (positions are given relative to the transcription start site [TSS]). The obtained sequence data have been submitted to the GenBank database (http://www.ncbi.nlm.nih.gov) under accession number U18812, and contain TATA box and binding sites for transcriptional factors C/EBP, Sp1 and Lp1. Methylation of the leptin promoter was analyzed by bisulfite sequencing. A DNA Purification Kit (cat. no. DN 1008, Biofuture Group Inc., Beijing, China) was used to isolate and purify DNA from the adipose tissue. Bisulfite conversion was performed using the Methylamp^TM^ DNA Modification Kit (cat. no. P-1001, Epigentek Group Inc., Brooklyn, NY). The converted DNA was amplified by nested PCR using Taq DNA Polymerase Master Mix (cat. no. KT201, Tiangen Biotech Inc., Beijing, China) and the following primers that were designed using Methprimer software: outer F, 5′-GAGTAGTTAGGTTAGGTATGTAAAGAG-3′; inner F, 5′-AGTTTTTTGTAGTTTTTTGTTTTTTG-3′; R, 5′-TAATAACTACCCCAATACCACTTAC-3′. The PCR conditions were as follows: 96°C for 10 minutes (min); 45 cycles of 96°C for 1 min, 51°C for 1 min and 72°C for 1 min; and 10 min at 72°C. The nested PCR was performed using the same conditions as the first step PCR. The PCR products were sequenced directly, and the DNA methylation was calculated as described by Lewin et al.^25^.

### Chromatin immunoprecipitation (ChIP)

Many promoter-associated proteins have been shown to be involved in the epigenetic regulation of gene transcription. We targeted the key histones and enzymes for which specific antibodies are available. The chromatin immunoprecipitation (ChIP) studies were performed using either prepared EpiQuik^TM^ kits (Epigentek Group Inc., NY), i.e., the Tissue Acetyl-Histone H3 and H4 ChIP Kit (cat. nos. P2012 and P-2013), Methyl-H3K4, -H3K9 and -H3K27 ChIP Kits (cat. nos. P-2009, P-2008 and P-2016), Tissue Methyl-CpG binding domain protein 2 (MBD2) ChIP Kit (cat. no. P-2018), or the general ChIP kit (Epigentek cat. no. P-2003) with specific antibodies for DNMTs (1, 3a and 3b) (cat. nos. ab13537, ab2850 and ab2851), HDACs (1, 2, 3, 4, 6, 7 and 9) (cat. nos. ab7028, ab7029, ab7030, ab79521, ab47181, ab50212 and ab59781), RNA Pol II (cat. no. ab5096) and CREB-binding protein (CBP) (cat. no. ab2832) (Abcam, Cambridge, MA, USA). Briefly, the procedure was performed as follows. Adipose tissue (20 mg) was cross-linked with 1% formaldehyde for 20 min and then homogenized. The homogenate was sonicated for 4 pulses of 15 seconds (sec) each at level 2 using the microtip probe of a Branson Digital Sonifier (Model 450, Branson Ultrasonics Corporation, Connecticut, USA), followed by a 40 sec interval on ice between each pulse, to generate fragments of genomic DNA ranging from 200 to 1000 bp in length. For the ChIP assays, equal amounts of treated chromatin were added to microwells containing immobilized antibody for the targeted protein or a negative control normal mouse IgG antibody. In addition, a small portion of treated chromatin, which was equal to 5% of the extracted genomic DNA, was used as the Input DNA to calculate the enrichment of the leptin promoter DNA after immunoprecipitation of the targeted proteins. After incubation for 90 min at 65°C to reverse the cross-links and elute the DNA, Fast-Spin columns were used for DNA purification.

The purified DNA was used for real-time PCR analysis of the leptin promoter. The sequence data for this gene are available through NCBI (http://www.ncbi.nlm.nih.gov) under the reference sequences NC_000072.5, covering nucleotides (nts) 29009221-29010220. The primers F- 5′ TCGAAGCAGGTGCATTCTGT 3′ and R- 5′ GGGCAACTTGTCTTCCTTTGG 3′ were used to generate an 80-bp amplicon (−431 to −510 relative to the TSS). The PCR conditions were as follows: 95°C for 10 min; 40 cycles of 95°C for 30 sec, 60°C for 30 sec and 75°C for 45 sec; and final steps of 7 min at 75°C and 2 min at 4°C. A melting curve was performed from 70°C to 95°C, and the signal was read every 0.5°C for 5 sec. The co-amplification of mouse GAPDH mRNA was performed in all samples including the ChIP and Input DNA. Each sample was normalized to the respective Input PCR product using the 2^–ΔC'T^ method. The data were then expressed as the ratio of ChIP to Input, which reflected the amount of the targeted proteins associated with the leptin promoter.

### Statistical analysis

One-way analysis of variance (ANOVA) tests were performed to compare the means between the two DIO groups and the control groups using SPSS Version 11.5 for Windows. Significant differences in the gene expression between the DIO groups and the control diet group were assessed using the Student-Newman-Keuls test, and comparisons between the DIO and n-3 PUFA DIO diet groups were determined using the Dunnett's test. In the case of unequal variance, the Games-Howell and Dunnett's T3 tests were used to examine the differences. Independent samples t was performed to compare the means between the DIO group and the control group during development of obesity. The Pearson test was used to assess the correlations between the leptin promoter methylation fraction and transcriptional expression. All values are expressed as the means ± SEM. Significant difference was set at P<0.05.

## Results

### Changes in the leptin mRNA level in obesity and the effects of n-3 PUFAs

As shown in Table 2, after 14 weeks of feeding, the average weight of the high-fat diet mice was similar between the DIO and n-3 PUFA DIO diet groups (38.09 ± 3.86 g and 38.02 ± 3.70 g, respectively) and was more than 50% higher than that of the control diet group (25.70 ± 1.08 g). The plasma leptin concentration in the DIO mice (8.59 ± 3.34 μg/L) was approximately 6-fold higher than that in the control mice (1.54 ± 1.13 μg/L), and consistently higher leptin mRNA expression was observed in the DIO mice (0.33 ± 0.07) compared with the control mice (0.02 ± 0.001) (P<0.01). The plasma leptin concentration (4.40 ± 2.00 μg/L) and adipose leptin mRNA levels (0.17 ± 0.05) in the n-3 PUFA DIO mice were significantly lower than those in the DIO mice (8.59 ± 3.34 μg/L and 0.33 ± 0.07) (P<0.05).

### Changes in DNA methylation of the leptin promoter in obesity and the effects of n-3 PUFAs

The methylated fractions in 9 of the 16 CpG sites at the leptin promoter were significantly higher by approximately 4% to 16% in the DIO mice compared with the control mice (P<0.05) (Table 3), including 3 CpG sites that are specific for the binding of the transcription factors SP1 and Lp1. However, dietary supplementation of n-3 PUFAs had no effects on the methylation of CpG sites in the leptin promoter.

### Changes in DNMTs, MBD2 and RNA Pol II binding at the leptin promoter in obesity and the effects of n-3 PUFAs

**Figure 1:**
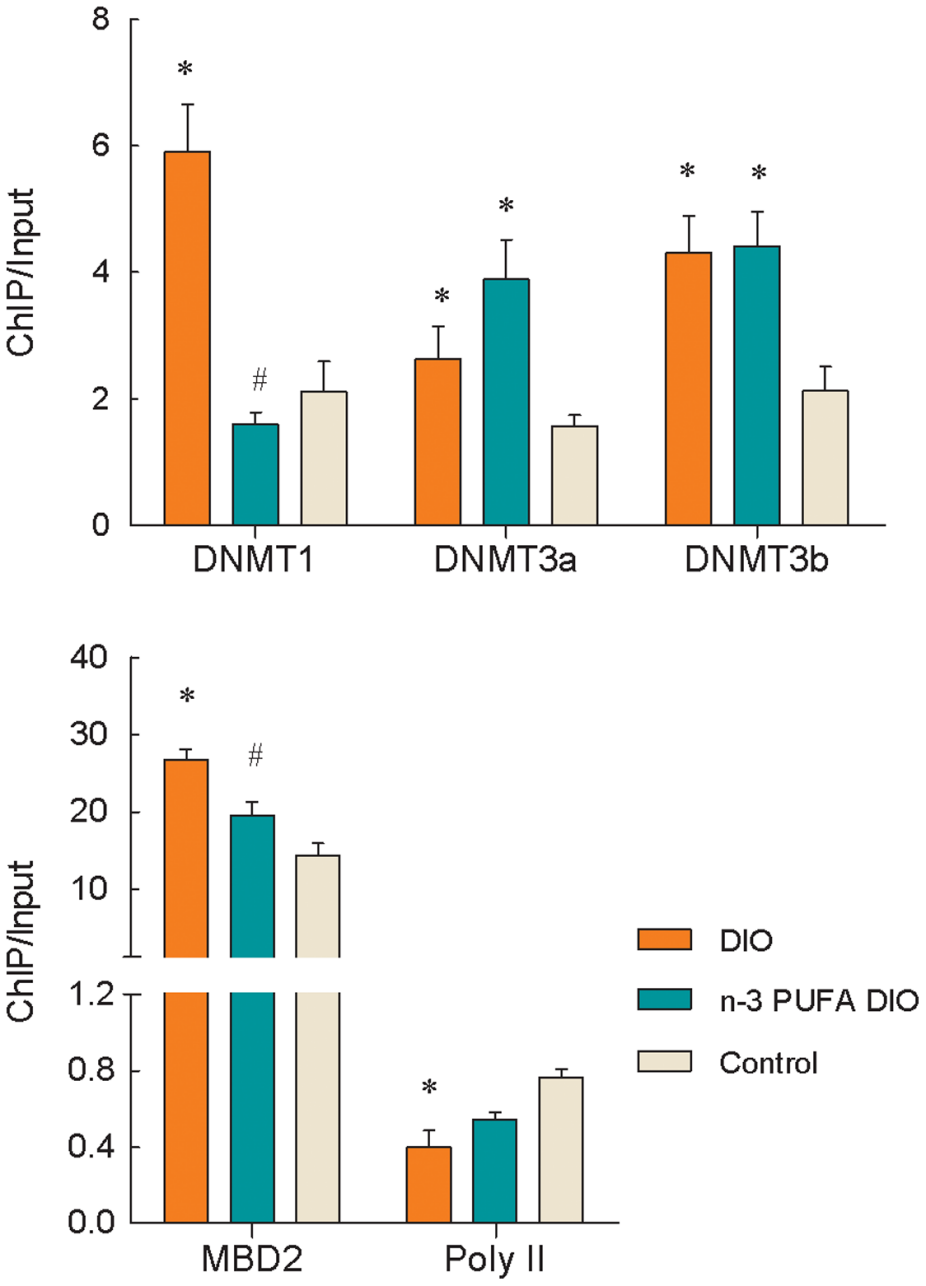
Changes in DNMTs, MBD2 and RNA Pol II binding at the leptin promoter in DIO mice. Genomic DNA was purified from mouse epididymal fat and was immunoprecipitated with antibodies specific for DNMT1, DNMT3a, DNMT3b, MBD2 and RNA Pol II. The immunoprecipitated DNA was used in PCR reactions to detect the presence of leptin promoter DNA. Normal mouse IgG was used as a negative control. The ratio of the PCR signal from the protein ChIP DNA to the signal from the total genomic DNA (Input) is plotted as an estimation of the protein levels. The values are expressed as the mean ± SEM; n = 12 in each group. * compared to the control diet, P<0.05; ^#^ compared to the DIO diet, P<0.005.

Significantly increased levels of DNMT1, DNMT3a, DNMT3b and MBD2 binding were observed at the leptin promoter in adipose tissue from the DIO diet group compared with tissue from the control mice (P<0.05), whereas significantly decreased levels of RNA Pol II binding were observed (P = 0.03) (Figure 1). Comparing the two groups of DIO mice, significantly reduced DNMT1 and MBD2 binding at the leptin promoter were observed in mice fed the n-3 PUFA DIO diet (P<0.005), whereas no changes were observed in the levels of DNMT3a, DNMT3b or RNA Pol II binding.

### Changes in histone acetylation associated with the leptin promoter in obesity and the effects of n-3 PUFAs

**Figure 2:**
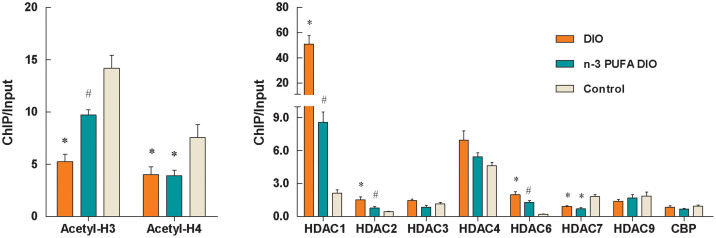
Changes in histone acetylation and HDAC binding at the leptin promoter in DIO mice. Genomic DNA was purified from mouse epididymal fat and was immunoprecipitated with antibodies specific for acetyl-H3, acetyl-H4, CBP and HDACs. The immunoprecipitated DNA was used in PCR reactions to detect the presence of leptin promoter DNA. Normal mouse IgG was used as a negative control. The ratio of the PCR signal from the protein ChIP DNA to the signal from the total genomic DNA (Input) is plotted as an estimation of the level of acetyl-H3, acetyl-H4, CBP or HDACs. The values are expressed as the mean ± SEM; n = 12 in each group. * compared to the control diet, P<0.01; ^#^ compared to the DIO diet, P<0.05.

In the DIO diet group, the levels of acetyl-H3 and acetyl-H4 at the leptin promoter were significantly lower and the binding of HDAC1, HDAC2 and HDAC6 was increased compared with the control diet group (P<0.01) (Figure 2). The introduction of n-3 PUFAs from fish oil in the diet significantly increased the H3 acetylation levels and reduced the binding of HDAC1, HDAC2 and HDAC6 to the leptin promoter (P<0.05). No differences in the binding of CBP to the leptin promoter were observed among the three different diet groups.

### Changes in histone methylation associated with the leptin promoter in obesity and the effects of n-3 PUFAs

**Figure 3:**
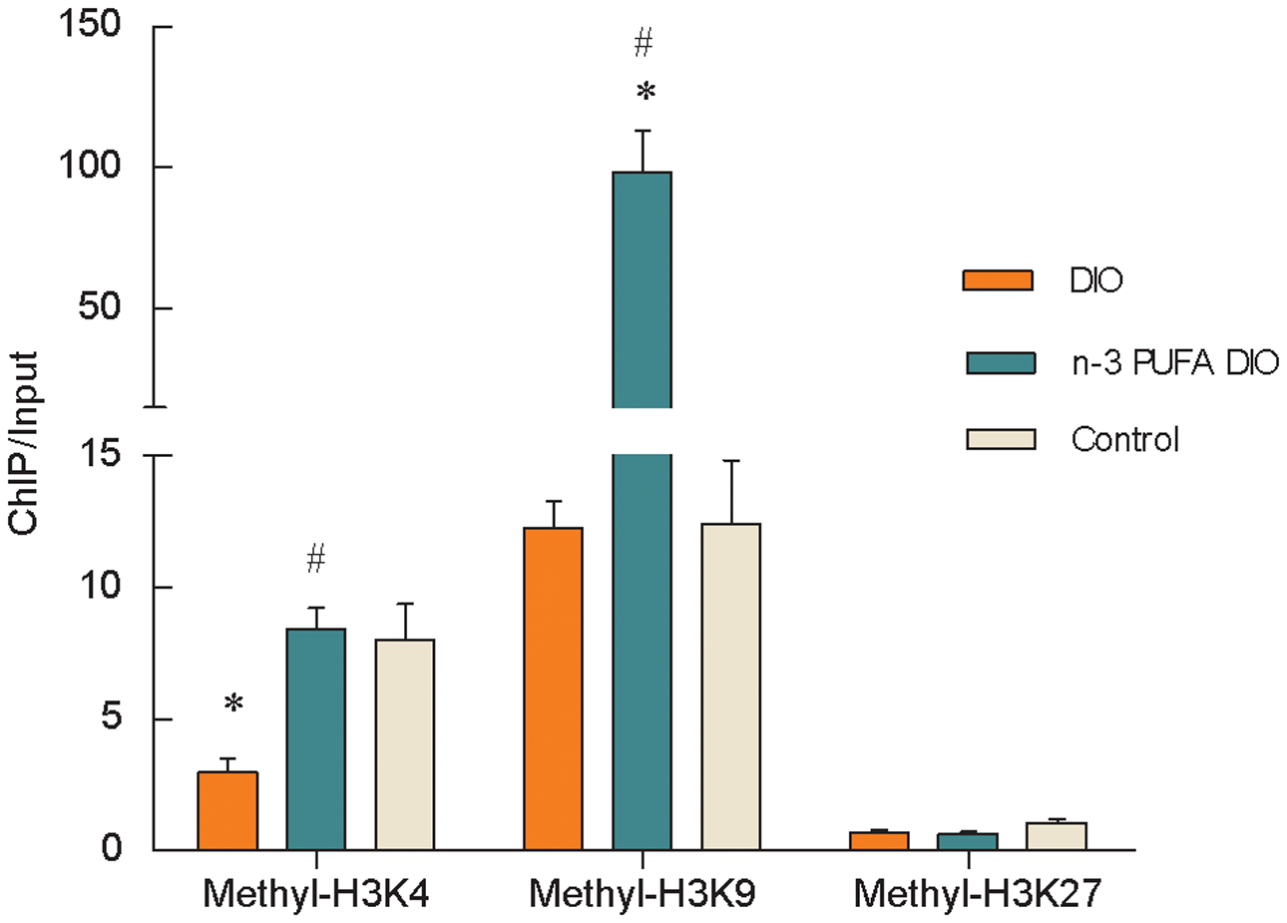
Changes in histone methylation at the leptin promoter in DIO mice. Genomic DNA was purified from mouse epididymal fat and was immunoprecipitated with antibodies specific for methyl-H3K4, methyl-H3K9 and methyl-H3K27. The immunoprecipitated DNA was used in PCR reactions to detect the presence of leptin promoter DNA. Normal mouse IgG was used as a negative control. The ratio of the PCR signal from the protein ChIP DNA to the signal from the total genomic DNA (Input) is plotted as an estimation of the level of methy-H3K4, H3K9 and H3K27. The values are expressed as the mean ± SEM; n = 12 in each group. * compared to the control diet, P<0.005; ^#^ compared to the DIO diet, P<0.001.

As shown in Figure 3, significantly reduced levels of methyl-H3K4 were observed at the leptin promoter in the DIO diet group compared with the control group (P<0.005), and no changes in the levels of methyl-H3K9 or methyl-H3K27 were observed. Comparing the two groups of DIO mice, methyl-H3K4 and -H3K9 levels at the leptin promoter were significantly increased in mice fed the n-3 PUFA DIO diet (P<0.001), which showed no difference in the level of methyl-H3K27.

### Association of the leptin promoter DNA methylation and gene transcription in the development of obesity

**Figure 4:**
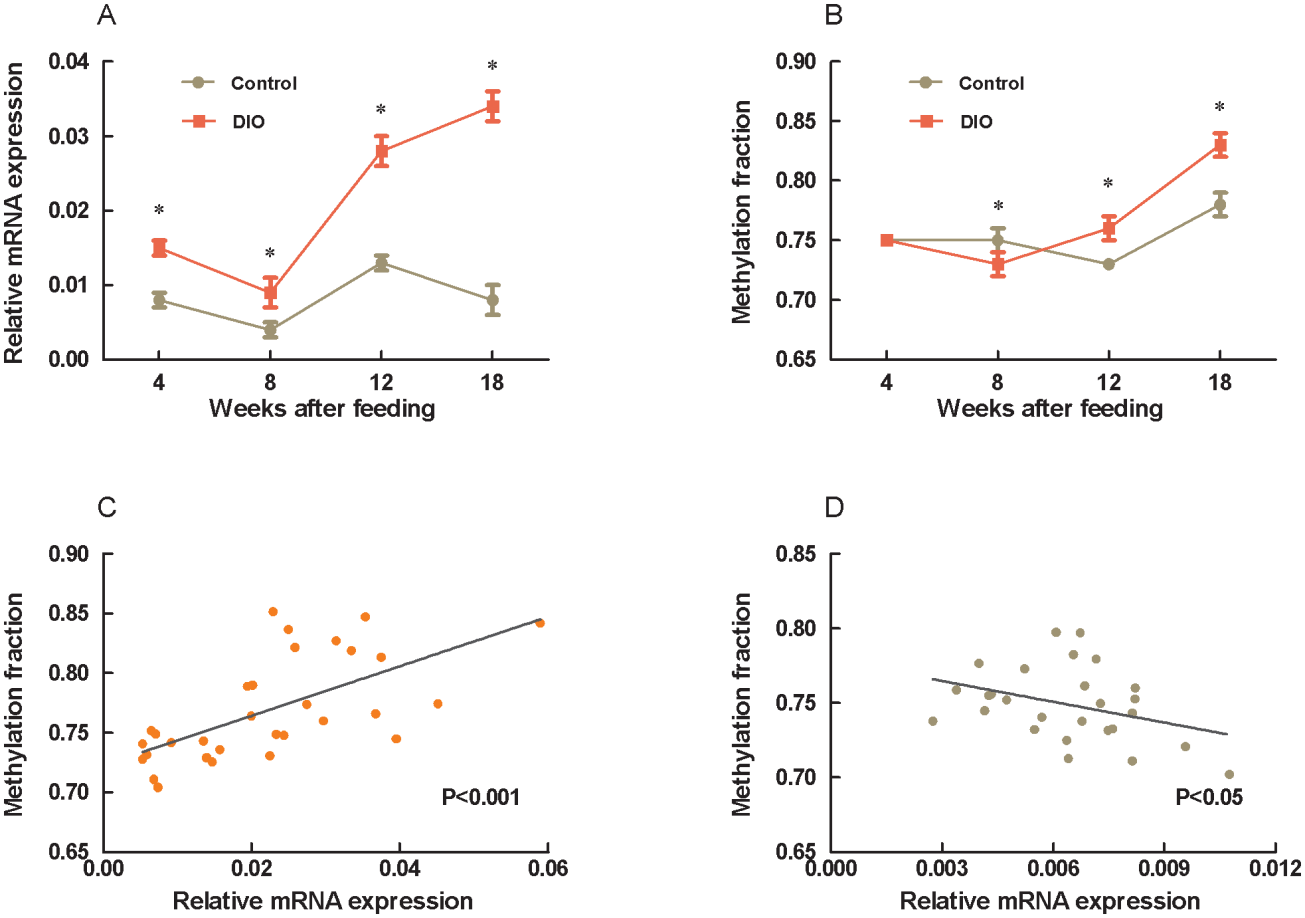
Changes in leptin mRNA expression, promoter methylation and their correlation in the development of obesity. Three to four-week-old C57BL/6J male mice were fed a DIO diet with a low-fat as control. At 4-, 8-, 12-, and 18-weeks after feeding respectively, mice were sacrificed and the epididymal fat was dissected. Gene transcripts in the epididymal fat were measured by real-time RT-PCR, and the promoter methylation fraction was calculated from the averaged methylation of the 16 CpG sites. A: mRNA expression; B: promoter methylation fraction; C: correlation between mRNA expression and promoter methylation in the DIO group; D: correlation between mRNA expression and promoter methylation in the control group. The values are expressed as the mean ± SEM; n = 15 in each group. * compared to the control group, P<0.05.

To further examine epigenetic regulation of the leptin expression, we detected CpG methylation at the leptin promoter and the mRNA expression during development of obesity. The averaged methylation fraction of 16 CpG sites at the leptin promoter was significantly reduced at 8 weeks and increased at 12 and 18 weeks after high fat diet feeding (P<0.001) with no changes at 4 weeks (Figure 4). Correlative analysis between methylation fractions and transcriptional mRNA expressions showed that a negative association in the control mice (r = 0.37, P<0.05) but a positive association in the DIO mice (r = 0.60, P<0.001) (Figure 4).

## Discussion

Epigenetic modifications are responsible for chromatin structure and stability as well as the modulation of tissue-specific gene expression, and epigenetics may be an important contributor to many chronic diseases including obesity^1^,^2^,^9^,^10^,^26^. The relationship between obesity and the epigenetic regulation of gene expression has been recently reported, and the methylation of the promoter regions of several genes has been demonstrated to change upon the development of obesity in animal models and humans^27^. DNA methylation is the only known modification that targets the DNA itself and is usually associated with gene silencing^2^,^28^. However, a negative correlation between methylation of the leptin promoter and leptin gene transcription was not observed in this current study. In the adipose tissue of the DIO mice, the methylation percentage at CpG sites and MBD2 binding to the leptin promoter was increased together with an increase in DNMT binding and a decrease in bound RNA Pol II; however, the leptin mRNA expression was unexpectedly increased. This apparent contradiction may be due to the involvement of other epigenetic modifications; DNA methylation at gene promoters regulates gene expression through a complicated mechanism involving multiple modifications, including the recruitment of HDAC and HMT complexes by the bound MBD2^18^,^19^.

**Figure 5:**
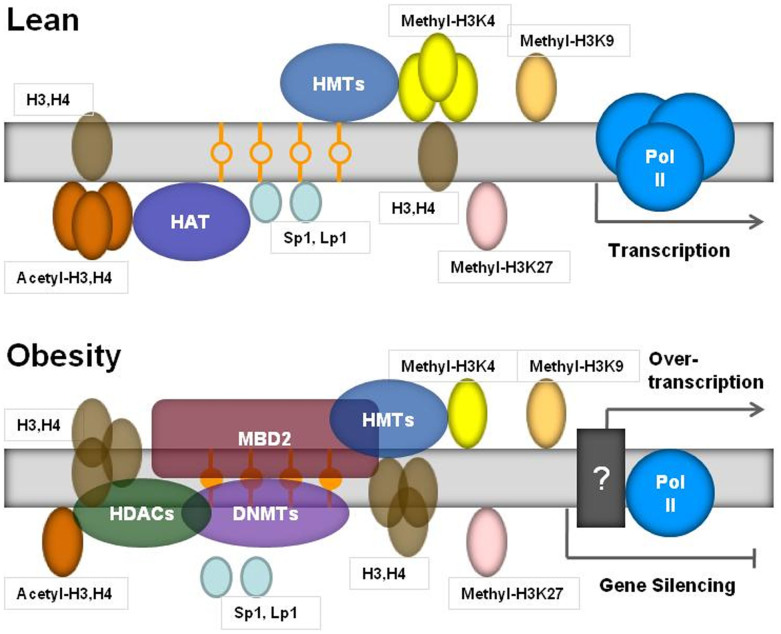
A schematic of the epigenetic mechanisms underlying leptin transcription in obesity. DNA methylation of the leptin promoter regulates the gene's transcriptional expression and changes with the development of obesity^12^,^13^,^14^,^15^,^16^. In this study, a more detailed description of the epigenetic modifications at the leptin promoter is elucidated for the obese state. With the development of obesity, leptin promoter methylation by DNMTs was increased together with MBD2 binding. Furthermore, the acetylation of histones H3 and H4, methylation of H3K4 and binding of RNA Pol II at the leptin promoter were reduced concomitant with an increase in HDAC (1, 2 and 6) binding. The complexes formed at this locus could tightly compact the nucleosomes, leading to a transcriptionally repressed state. However, the leptin mRNA expression was increased, not decreased, in obesity. Therefore, the epigenetic modifications at the leptin promoter, which may occur as one component of a feedback regulation mechanism due to the increased gene expression, are not sufficient to return the gene expression to a normal level. The unknown mechanisms (black box) underlying leptin transcription in obesity remain to be explored.

Not only does epigenetic modification occur through the cytosine methylation of DNA, histones are also subject to many modifications, including methylation, acetylation, phosphorylation and ubiquitination. Histone modifications influence chromatin structure and, hence, the ability of critical DNA-associated regulatory proteins that control transcription, replication, recombination and repair to gain access to the DNA^9^,^19^. Histone acetylation, controlled by HATs (SRCs, CBP and P300) and HDACs, which add or remove acetyl groups from target histones, respectively, plays key roles in modulating chromatin structure and function^29^,^30^. Acetylation generally promotes gene transcription by relaxing chromatin structure, thereby facilitating access of the transcriptional machinery to DNA target sequences. This effect is counterbalanced by histone deacetylation, which favors chromatin condensation and transcriptional repression^6^,^7^. Recent studies have revealed that changes in histone modification are a key component of the epigenetic network controlling adipogenesis and energy homeostasis^8^,^9^,^10^,^31^. Although increasing evidence indicates that HDACs may activate genes by removing the acetyl group and resetting the chromatin structure, their roles in repression are still supported by the HDAC-associated prevention of RNA Pol II binding at promoters^6^,^7^. In this sense, our findings that three HDACs (1, 2, and 6) associated with the leptin promoter were increased, together with the hypoacetylation of H3 and H4 and loss of Pol II binding in DIO mice, suggests that these changes may lead to the inactivation of the leptin gene. In addition, H3K4 hypomethylation at the leptin promoter may lead to decreased leptin mRNA levels due to the downregulation of gene transcription^7^,^8^. However, we found that leptin mRNA expression in the adipose tissue of the DIO mice was increased instead of reduced, leading to a higher leptin concentration in the plasma. These data further emphasize the contradiction between the epigenetic modifications and transcription in the regulation of the leptin gene. Furthermore, we found that DNA methylation of the leptin promoter varied with the process of obesity. The leptin promoter methylation fraction in DIO mice was decreased at initial time by high-fat feeding, and increased after 12-week feeding,compared to control with the low-fat diet. Further analysis showed that the promoter methylation level and gene transcription had a negative association in control mice but a positive association in DIO mice. Therefore, a schematic model of the mechanism underlying leptin gene expression in obesity is shown in Figure 5. To our knowledge, the changes in DNA methylation, histone modifications and RNA Pol II binding at the leptin promoter may result from feedback due to the increased gene expression, and these epigenetic modifications are not sufficient to return the gene expression to normal.

Epidemiological studies have demonstrated that increased n-6 PUFAs and/or decreased n-3 PUFAs in the modern human diet are risk factors for the increased prevalence of chronic non-communicable diseases in modern society including obesity^32^,^33^. The beneficial role of n-3 PUFAs in the pathogenesis of obesity is attributed to their regulation of fatty acid synthesis and oxidation. In addition, n-3 PUFAs control the differentiation and proliferation of adipocytes through the modulation of gene transcription, messenger RNA processing and posttranslational protein modifications^20^,^34^. In this study, we found that n-3 PUFA supplementation in high-fat diets reduced mRNA expression of the leptin gene in adipose tissue, which is consistent with other reports^21^,^22^,^23^. Epigenetic modifications may contribute to the underlying regulatory mechanisms because methyl groups are required for the conversion of PE-DHA to PC-DHA. Upon deficiency of tissue and cellular DHA, excess methyl groups will be available for other trans-methylation reactions such as DNA and histone methylation, leading to altered chromatin remodeling and gene expression^35^. In this present study, dietary n-3 PUFAs did not affect the methylation level of CpG sites at the leptin promoter in the adipose tissue of the DIO mice, although DNMT1 and MBD2 binding were reduced, and the levels of methyl-H3K4 and acetyl-H3 increased concomitant with the reduced binding of HDAC1, HDAC2 and HDAC6. These data suggest that n-3 PUFAs seemed to antagonize leptin promoter modifications induced by DIO diet. Unexpectedly and interestingly, methylation of H3K9 was greatly promoted by n-3 PUFAs in obesity. It has been reported that methylated H3K9 provides a binding platform for heterochromatin protein 1, which associates with a variety of other factors including HDACs, transcriptional repressors and chromatin remodeling enzymes, and finally influence heterochromatin formations inactivating transcriptional state^36^,^37^,^38^. This may override the other modifications of the promoter and play an important role in mediating the down-regulation of leptin expression with n-3 PUFAs. Nonetheless, a detailed analysis of the regulation of various factors associated with the leptin promoter after supplementation of n-3 PUFAs is needed in the future.

In summary, the results of this study have revealed that important epigenetic changes occur in the leptin promoter of DIO mice by either directly inhibiting enzymes that catalyze DNA methylation and histone modifications or altering the availability of substrates necessary for these enzymatic reactions^39^. These epigenetic modifications may serve a feedback role to maintain leptin concentrations within a normal range. The regulation of leptin expression by n-3 PUFAs in dietary fish oil is mediated, at least in part, by epigenetic factors, such as MBD2 and histone modifications. These findings provide insights regarding new targets, including the potential epigenetic regulation of key metabolic genes, which may be important for preventing or treating obesity.

## Figures and Tables

**Table 1:** Composition of the animal diets

	DIO	n-3 PUFA DIO	Control
Components (g/kg)			
Lard	318	318	20
Sunflower oil	35	-	25
Fish oil	-	35	-
Fatty acid composition (% of fat)			
Σ Saturated	41.83	44.01	27.22
Σ Monounsaturated	41.62	42.81	30.67
18:2n-6	15.51	8.59	41.14
20:4n-6	0.94	0.62	0.97
22:4n-6	-	0.9	-
Σ n-6 PUFAs	16.55	10.11	42.11
18:3n-3	0.12	0.11	0.09
20:5n-3	-	1.85	-
22:6n-3	-	1.11	-
Σ n-3 PUFAs	0.12	3.07	0.09
Total energy (kcal%)			
Fat	60	60	10
Protein	20	20	20
Carbohydrate	20	20	70

**Table 2:** Changes in body weight and leptin expression in plasma and adipose tissue of DIO mice. Beginning at age 4–5 weeks, C57BL/6J male mice were fed the DIO, n-3 PUFA DIO or control diet for 14 weeks. The data are expressed as the mean ± SEM; n = 12 in each group

	DIO diet	n-3 PUFA DIO diet	Control diet
Body weight (g)	38.09 ± 1.27*	38.02 ± 1.23*	25.70 ± 0.36
Plasma leptin (μg/L)	8.59 ± 1.11*	4.40 ± 0.66* #	1.54 ± 0.38
Leptin mRNA in fat	0.33 ± 0.03*	0.17 ± 0.02* #	0.02 ± 0.001

*compared to the control diet, P<0.05.

^#^compared to the DIO diet, P<0.05.

**Table 3:** Quantitative methylation analysis of the leptin promoter. Beginning at age 4–5 weeks, C57BL/6J male mice were fed a DIO, n-3 PUFA DIO or control diet for 14 weeks. Genomic DNA isolated from epididymal fat was analyzed for the methylation of 16 CpG sites (*a, b, … p*) at the indicated positions in the leptin gene promoter spanning nucleotides -321 to -1 relative to the TSS. The methylation fraction was calculated from the amplitude of cytosine and thymine within each CpG dinucleotide, C/(C+T). The result for each CpG site is represented as the mean ± SEM determined from 12 mice in each group.

-321agccttctgtagcctcttgctccctgcggtgctggaagcaccatcccaagggacccgtccttaaactaccg**a**ctgctcagtagctgctggccg**b**gacctcg**c**aggattaccg**d**gctcataccaagcg**e**cccccaaacttgcactcg**f**agggcg**g**cg**h**gctgaagttctccctcg**i**aggcg**j**cctagaatggagcactaggttgctgctgccactgttgctggcccg**k**ctgggtggggcg**l**gga**Sp1**gttggcg**m**ctcg**n**caggg**Lp1**actggggctggccg**o**gacagttgcg**p**caag**C/EBP**tggcactggggcagttataa**TATA-box**gaggggcaggcaggcatggagccccg -1 (TSS)																	
*Diets*	*CpG sites*																
*a*	*b*	*c*	*d*	*e*	*f*	*g*	*h*	*i*	*j*	*k*	*l*	*m*	*n*	*o*	*p*	*total*	
DIO	0.66 ± 0.06	0.80 ± 0.03*	0.64 ± 0.06	0.72 ± 0.05*	0.81 ± 0.05*	0.68 ± 0.04*	0.79 ± 0.09	0.84 ± 0.03*	0.67 ± 0.07	0.80 ± 0.05*	0.77 ± 0.04	0.81 ± 0.05*	0.82 ± 0.04*	0.78 ± 0.05*	0.90 ± 0.06	0.93 ± 0.03	0.78 ± 0.04*
n-3 PUFA DIO	0.64 ± 0.05	0.75 ± 0.03	0.61 ± 0.05	0.66 ± 0.05	0.76 ± 0.04	0.66 ± 0.05	0.77 ± 0.04	0.82 ± 0.03	0.65 ± 0.06	0.77 ± 0.04	0.77 ± 0.04	0.79 ± 0.05	0.80 ± 0.05	0.78 ± 0.07	0.88 ± 0.04	0.94 ± 0.02	0.75 ± 0.04
Control	0.61 ± 0.05	0.74 ± 0.03	0.61 ± 0.04	0.64 ± 0.04	0.73 ± 0.06	0.59 ± 0.19	0.74 ± 0.05	0.79 ± 0.05	0.67 ± 0.11	0.76 ± 0.04	0.75 ± 0.03	0.74 ± 0.04	0.79 ± 0.03	0.74 ± 0.03	0.88 ± 0.03	0.94 ± 0.01	0.72 ± 0.08

*compared to the control group, P<0.05.
